# The effect of musical-animated toys and audiobooks on fear and pain in the tracheostomy care of children in the palliative care

**DOI:** 10.1590/1806-9282.20241023

**Published:** 2024-12-02

**Authors:** Gamze Akay, Türkan Kadiroğlu, Fatma Güdücü Tüfekci, Aysun Öncer, Döne Kiliç Bulut

**Affiliations:** 1 Artvin Coruh University, Vocational School of Health Services, Department of Pediatric Nursing – Artvin, Turkey.; 2 Atatürk University, Faculty of Nursing, Department of Pediatric Nursing – Erzurum, Turkey.; 3 Erzurum City Hospital, Faculty of Medicine, Department of Palliative Care – Erzurum, Turkey.

**Keywords:** Pediatric, Palliative care, Pain, Fear

## Abstract

**OBJECTIVE:**

The study aimed to evaluate the effects of musical-animated toys and audiobooks on the fear and pain in the tracheostomy care of children in the palliative care clinic.

**METHODS:**

The study design was a single-center, single-arm, crossover-controlled study. The sample consisted of 16 children who were 3–6 years old. Musical-animated toys and audiobooks were used to divert the children’s attention during tracheostomy care. The children whose control data were collected on the first day were shown musical-animated toys on the second day and listened to an audiobook a week later. The children were video-recorded during the interventions.

**RESULTS:**

The children who received musical-animated toy and audiobook interventions during and after tracheostomy care expressed less pain than those in the control group, and their fear levels were less during the care.

**CONCLUSION:**

Audiobook and musical-animated toy interventions were effective in reducing children’s procedure-related fear and pain during tracheostomy care in the pediatric palliative care clinic.

## INTRODUCTION

Children receiving pediatric palliative care often experience significant loss of neurological and physical functions early in life, leading to complete dependency on caregivers and prolonged hospitalizations^1^. Hospital treatments include painful procedures such as endotracheal aspiration, dressing changes, catheter operations, intubation, and extubation. Among these, tracheostomy care is noted as distressing and painful^2^.

Children undergoing intensive treatment often experience significant fear and stress due to frequent painful procedures. These procedures compromise physiological stability, leading to increased blood pressure and systemic effects such as immune suppression and delayed healing. It is crucial to implement nursing interventions aimed at reducing pain and fear and supporting the children’s overall well-being, treatment, and recovery^3^.

Various non-pharmacological methods are used to alleviate pain and fear in children. Distraction techniques, such as engaging the child with games, stories, or other activities, can effectively lessen the perception of pain during procedures. Redirecting attention to pleasant sensations, like listening to music or being in a comforting environment, also helps reduce perceived pain^3,4,5,6^.

Children aged 3–6 are particularly responsive to visual and auditory stimuli, showing a keen interest in exploring their senses and learning^5,6^. Stimuli like colorful pictures, animations, videos, music, audio stories, and rhymes captivate their attention. Toys that combine music and animation, along with audiobooks, are examples of such stimuli. While pharmacological methods are used in managing pain during tracheostomy care^7,8^, there is a lack of studies focusing on distraction methods during this procedure for children. Non-pharmacological approaches should be prioritized for pain management^9^. This study aims to determine the effects of musical-animated toys and audiobooks on pain, fear, and physiological parameters in the tracheostomy care of children in the palliative care clinic.

## METHODS

### Study design and sample

**Figure 1:**
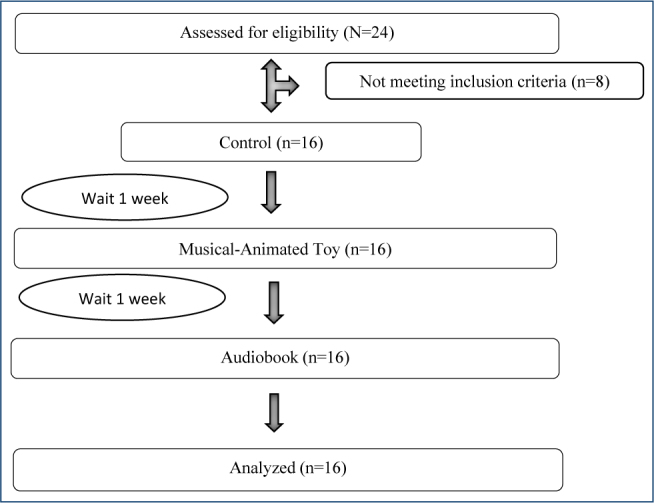
Study flow chart.

This single-center, single-arm, crossover-controlled study was conducted in the pediatric palliative care clinic of a central public hospital in eastern Turkey. The study was recorded within the framework of the CONSORT Report (2017) (Figure 1).

The study population consisted of the children who received inpatient treatment at the pediatric palliative care clinic between November 2023 and December 2023 and met the inclusion criteria of the study. The inclusion criteria were as follows: (i) being between 3 and 6 years of age, (ii) receiving treatment in a pediatric palliative care clinic, (iii) having a tracheostomy, and (iv) having no vision or hearing anomalies. The exclusion criteria were as follows: (i) the desire to leave the study by the family and (ii) the children’s general health condition deteriorating. The entire population was examined without resorting to the sampling method, and the study was completed with 16 children who met the inclusion criteria.

Power analysis: The post-hoc power analysis was used to determine the adequacy of the sample size of the study. It was determined in the power analysis that the effect size was 0.75 and the power was 0.99 at the 0.05 significance level and 95% confidence interval.

### Data collection tools

Descriptive data form: This form was created by the researchers following a review of the literature^10,11^. There are records of physiological parameters including pulse, respiratory rate, blood pressure, and SpO_2_ values.

FLACC (face, legs, activity, crying, consolability) pain scale: This scale was developed by Merkel et al. for assessing pain in children aged 2 months to 18 years who are unable to verbally communicate their pain. This scale measures five behavioral categories: facial expressions, leg movements, activity level, crying, and the ability to be comforted. Each category is rated on a scale from 0 to 10, where 0 indicates no pain, 1–3 indicates mild pain, 4–6 indicates moderate pain, and 7–10 indicates severe pain^12^.

Children’s Fear Scale (CFS): This scale was developed by McMurtry et al. in 2011 and is used to detect fear and anxiety levels in children. The scale consists of five statements with 0 corresponding to no fear and no anxiety and 4 to severe fear and anxiety. The scale can be evaluated by the researcher or by the parent^13^.

Musical-Animated Toy: The musical-animated toy was chosen based on the children’s ages and current conditions, with input from experts in child development, child health, and music therapy. Approved by the Turkish Standards Institute, the toy, resembling a cartoon character, features flashing lights, animated movements, and dancing to music, effectively capturing children’s attention.

While the toy appeals to all ages, it especially interests children aged 3–6 years. It was placed next to the child’s bed for easy visibility. Care began once the child was focused on the toy. To reduce the risk of infection, a special musical toy was bought for each child and given to the child after the study.

Audiobook: The audiobook was chosen based on the children’s ages and conditions, with input from child psychologists and health specialists. It was uploaded to each child’s room TV before tracheostomy care, ensuring it was long enough to cover the 5 min before and during the procedure.

### Data collection

The order of intervention was determined by lot. The lottery was conducted by a nurse independent of the study using a closed, opaque envelope. The participating children were assigned to the following groups in the study: 1—control, 2—music-animated toy, and 3—audiobook. Children were video-recorded to assess their pain and fear during tracheostomy care.

Piaget proposed that children actively interact with their environment and go through age-specific developmental stages. In the preoperational stage (ages 3–6 years), children use symbolic and intuitive reasoning rather than logical thinking, focusing on images and not yet understanding the conservation of quantity^14^. The study implemented a 1-week washout period between interventions.

The children were video-recorded during the interventions. A video camera was placed at the foot of the bed, slightly above the child’s body level, to capture every movement, gesture, and expression.

The data of the study were collected by the researcher at the same care hour with the same nurse to exclude individual differences during tracheostomy care. The patients were never left alone throughout the interventions, and necessary safety precautions were taken. The beds of the patients were brought to the appropriate level by the researcher and the side where the intervention would be performed was lowered.

The descriptive data of the children included in the study were obtained from the files at their bedsides. Physiologic parameters of the children were recorded with bedside monitors used in the clinical routine. The pain and fear levels of the children were evaluated three times, 5 min before, during, and 5 min after tracheostomy care. These assessments were made separately by three experts who reviewed the video recordings.

### Data analysis

Mean, standard deviation, median, minimum, maximum, percentage, number, kurtosis, and skewness coefficients, Friedman’s analysis, the Kruskal-Wallis test, least significant difference (LSD), and the Dunnett C test were used in the analysis of the data. Intra- and interobserver agreement of the video recordings was analyzed by intraclass correlation coefficient (ICC). For FLACC, it was found to be 0.906 before care, 0.756 during care, and 0.905 after care. The ICC for CFS was determined to be 0.825 before treatment, 0.758 during treatment, and 0.856 after treatment. There was a very good level of agreement between the experts, who were the judges of the video recordings.

### Ethical considerations

The study was approved by the Ataturk University Faculty of Medicine Ethics Committee (Date: 26.10.2023 Number: B.32.ATA.0.01.00/768). An official authorization letter was secured from the hospital where the research procedures were carried out. The study was registered at https://clinicaltrials.gov/ (ClinicalTrials.gov Identifier: NCT06121440).

## RESULTS

A total of 68.8% of the participants were males and 43.8% were diagnosed with SMA Type 1. The mean age of the participants was 3.94±1.06, the mean duration of hospitalization was 33.19±19.15 months, and the duration of the current diagnosis was 6.56±1.11 years (Table 1).

**Table 1:** Descriptive characteristics of children.

Characteristics	n	%
Gender		
Girl	5	31.3
Boy	11	68.8
Diagnosis		
SMA Type 1	7	43.8
Cerebral palsy	2	12.5
Pierre Robin syndrome and cleft palate	1	6.3
Hidrosefali	1	6.3
Metabolic disease	2	12.5
Bronchopulmonary dysplasia	1	6.3
Meningomyelocele	1	6.3
Tanatoforik displazi Tip 2	1	6.3
Continual variables (n=16)	Mean±SD	
Age (year)	3.94±1.06	
Hospitalization duration (month)	33.19±19.15	
Diagnosis duration (year)	6.56±1.11	

SD: standard deviation.

Before the procedure, the differences in the means of pain, fear, and physiological parameters between the control, musical-animated toy, and audiobook groups were not statistically significant (p>0.05) (Table 2).

**Table 2:** Comparison of pain, fear, and physiological parameters in groups.

		Control		Musical-animated toy		Audiobook		Test	p
		Mean	SD	Mean	SD	Mean	SD		
FLACC	Before	1.38	1.45	2	1.9	2.31	2.09	F=1.085	0.347
	During	4.31	1.08	1.69	0.95	2.13	1.41	F=23.490	≤**0.001**
	After	2.56	1.09	1.06	0.57	0.88	0.72	F=20.112	≤**0.001**
CFS	Before	1.94	0.77	2	1.32	2.31	1.08	F=0.555	0.578
	During	3.63	0.5	1.38	0.81	1.19	0.98	F=47.416	≤**0.001**
	After	0.94	0.68	0.44	0.73	0.5	0.63	F=2.560	0.089
Pulse	Before	109.81	20.52	104.13	17.2	112.88	22.74	F=0.767	0.47
	During	124.75	23.04	104.5	24.32	106.44	23	F=3.630	**0.035**
	After	107.19	20.92	103.19	18.13	102.88	18.05	F=0.254	0.777
Respiratory	Before	28.13	2.47	28.63	3.56	28.75	2.41	x^2^_KW_=0.474	0.789
	During	31.25	2.72	28.13	4.22	26.88	2.06	F=8.263	≤**0.001**
	After	30.63	3.63	28.38	3.03	27	1.93	F=6.159	**0.004**
SpO_2_	Before	98.06	2.54	99.06	1.81	97.75	2.82	x^2^_KW_=3.285	0.193
	During	97	2.85	98	2.48	96.81	3.47	x^2^_KW_=1.360	0.507
	After	98.31	1.35	98.31	1.14	97.94	2.17	x^2^_KW_=0.185	0.912
Systolic blood pressure	Before	106.56	14.57	104.31	14.94	103.81	26.43	F=0.091	0.913
	During	115.88	15.06	102.31	13.46	104.19	10.92	F=4.916	**0.012**
	After	101.13	13.06	104.13	9.59	103.88	8.21	F=0.403	0.671
Diastolic blood pressure	Before	67	13.49	63.19	10.86	68.38	11.09	x^2^_KW_=0.893	0.64
	During	72.25	13.57	64.88	9.66	71.5	15	F=1.574	0.218
	After	64.44	10.94	64.94	10.25	63.81	9.03	F=0.050	0.951

FLACC: face, legs, activity, crying, consolability pain scale; CFS: children’s fear scale; F: Friedman’s analysis; KW: Kruskal-Wallis; SD: standard deviation. Bold indicates p<0.05.

During the procedure, there were statistically significant differences in the pain (p≤0.001), fear (p≤0.001), pulse (p=0.035), respiration (p≤0.001), and systolic pressure (p=0.012) means among the groups. The control group had higher means of pain (4.31±1.08; moderate level), fear (3.63±0.50; moderate level), pulse (124.75±23.04), respiration (31.25±2.72), and systolic pressure (115.88±15.06) compared to the musical-animated toy and audiobook groups (Table 2).

After the procedure, there were statistically significant differences in the pain (p≤0.001) and respiratory (p=0.004) means among the groups. The pain score (2.56±1.09) and respiratory (30.63±3.63) mean of the control group was higher than the mean of the musical-animated toy and audiobook groups (Table 2).

## DISCUSSION

This study is a single-center, single-arm, crossover-controlled trial. This design was chosen due to the low number of pediatric palliative care (PPC) patients. In a crossover design, applying interventions to the same group eliminates errors caused by group differences, making comparisons more accurate. A disadvantage of the crossover design is that the effect from one period can influence the next intervention (transmitted effect). In our study, a 1-week washout period was implemented to account for the children’s age-related characteristics, thus preventing the transmitted effect.

Tracheostomy is of vital importance in ensuring airway patency. However, patients also experience problems such as infection, pain, skin problems, incorrect position of the tube, and tracheal stenosis during this procedure. To prevent problems that may arise, the care process of patients (aspiration, cannula cleaning, stoma care, etc.) must be continued effectively^15^. In our study, before tracheostomy care, there was no significant difference in pain and fear between the intervention and control groups, which is a known and expected result^16,17^. In the study, children experienced moderate pain and fear during tracheostomy care. The moderate level of pain and fear reveals the need for effective pain management in tracheostomy care.

During the procedure, using an audiobook and a musical-animated toy significantly reduced pain and fear in the intervention groups compared to the control group. This aligns with other studies showing that distraction techniques effectively reduce children’s pain^9,18,19^ and fear^4,5,9^. The dissemination of distraction techniques in clinical practice may make a significant contribution to reducing procedural pain and fear in children. Their use, especially during procedures such as tracheostomy care in pediatric patients, may have positive effects on the general well-being of children.

Pain and fear impact children’s physiological parameters, notably blood pressure, pulse, and respiration^20^. In this study, pulse, respiration, and systolic blood pressure remained normal before the tracheostomy procedure. However, the musical-animated toy and audiobook interventions in the experimental groups positively influenced these parameters compared to the control group. Previous studies on pulse^11,20,21^, respiration^11,20,21^, and blood pressure^11,22,23,24^ support these findings, showing that these interventions are effective distraction methods that positively affect physiological parameters during tracheostomy care.

Using a musical-animated toy and an audiobook significantly reduced pain in the intervention groups after the procedure when compared to the control group. Other studies evaluating pain with similar distraction methods support these findings^16,19,2,25^.

## CONCLUSION

Audiobooks and musical-animated toys, which are distraction attempts used among non-pharmacological pain relief methods, reduced the pain and fear significantly during the procedure and pain after the procedure in children receiving PPC compared to the control group, which indicates that these interventions are effective care interventions. Audiobook and musical-animated toy initiatives can positively influence children’s feelings toward health care and make future painful procedures easier for them.
